# Do microplastics play a role in the pathogenesis of neurodegenerative diseases? Shared pathophysiological pathways for Alzheimer’s and Parkinson’s disease

**DOI:** 10.1007/s11010-025-05428-3

**Published:** 2025-11-18

**Authors:** Alexander Chi Wang Siu, Keshav Raj Paudel, Gurjeet Singh, Gaurav Gupta, Sachin Kumar Singh, Dinesh Kumar Chellappan, Gabriele De Rubis, Suhrud Pathak, Brian Gregory George Oliver, Kamal Dua, Muralikrishnan Dhanasekaran

**Affiliations:** 1 Discipline of Pharmacy, Graduate School of Health, University of Technology Sydney, Ultimo, NSW 2007 Australia; 2 Centre for Inflammation, School of Life Sciences, Faculty of Science, Centenary Institute and University of Technology, Sydney, NSW 2007 Australia; 3 Woolcock Institute of Medical Research, Macquarie University, Sydney, NSW Australia; 4 Uttaranchal Institute of Pharmaceutical Sciences, Uttaranchal University, Dehradun, India; 5 Centre for Research Impact & Outcome, Chitkara College of Pharmacy, Chitkara University, Rajpura, 140401 Punjab India; 6 Centre of Medical and Bio-allied Health Sciences Research, Ajman University, Ajman, 346 United Arab Emirates; 7 School of Pharmaceutical Sciences , Lovely Professional University, Phagwara, India; 8 Faculty of Health, Australian Research Centre in Complementary and Integrative Medicine, University of Technology Sydney, Ultimo, NSW 2007 Australia; 9 Department of Life Sciences, School of Pharmacy, International Medical University, Bukit Jalil, Kuala Lumpur, 57000 Malaysia; 10 Department of Drug Discovery & Development, Harrison College of Pharmacy, Auburn University, 3306B Walker building, Auburn, AL 36849 USA; 11 School of Life Sciences, University of Technology Sydney, Ultimo, NSW 2007 Australia; 12 Graduate School, Auburn University, Auburn, AL 36849 USA

**Keywords:** Microplastics, Alzheimer’s disease, Parkinson’s disease, Neurodegenerative diseases, Plastic environmental pollution, Neuro-pathophysiology

## Abstract

The widespread presence of microplastics (MPs) in the environment has raised significant concerns about their potential impact on human health. As of 2023, the Ocean Conservancy estimates that adults may ingest up to 121,000 MPs annually. While the majority of these particles are cleared from the body, a small fraction can persist, as MPs are non-biodegradable and resist breakdown, posing long-term health risks that remain poorly understood. This review explores the emerging link between MP exposure and the development of neurodegenerative diseases, particularly Alzheimer’s disease (AD) and Parkinson’s disease [1]. MPs appear capable of triggering neurotoxic pathways, including activation of resident immune cells in the brain, oxidative stress, blood–brain barrier (BBB) disruption, mitochondrial dysfunction, and neuronal damage, which may contribute to neuroinflammation and disease progression. Specifically, six MP-related mechanistic pathways associated with AD were identified: BBB disruption, chronic inflammation, oxidative stress and ROS generation, mitochondrial dysfunction, impaired autophagy and proteostasis, and epigenetic alterations. Similarly, six pathways were implicated in PD: BBB disruption, oxidative stress in dopaminergic neurons, mitochondrial dysfunction, microglial-driven neuroinflammation, α-synuclein aggregation, and gut–brain axis [2] disruption. Ultimately, our findings underscore the urgent need for further research into the neurological consequences of chronic MP exposure in humans and highlight the importance of strengthening global policies to curb plastic pollution and mitigate its long-term health risks.

## Introduction

 Following Professor Richard Thompson’s groundbreaking discovery of microplastic (MP) particles in 2004, research into their environmental and biological impacts has expanded significantly [3, 4]. As of 2023, the Ocean Conservancy estimates that an average adult may ingest up to 121,000 MPs annually [5]. While the human body is generally capable of eliminating over 90% of ingested MPs, the small fraction that remains can persist and potentially exert chronic, long-term effects on human health—effects that are still not fully understood [5].

Alarmingly, MP pollution has now been detected in a wide range of environments and biological systems, including human food, breast milk, and even in some of the planet’s most remote locations, such as the Arctic, the summit of Mount Everest, and the depths of the Mariana Trench [6, 7]. Moreover, in samples of snow and stream water taken at different elevations on the mountain, the study form Napper et al. found microplastics. The sample collected at 8,440 m above sea level had the greatest concentration of microplastics. The majority of the microplastics found were polyester fibers, most likely from climbers’ gear and apparel. According to the paper, future expeditions might reduce microplastic contamination by utilizing technical improvements [8].

**Fig. 1:**
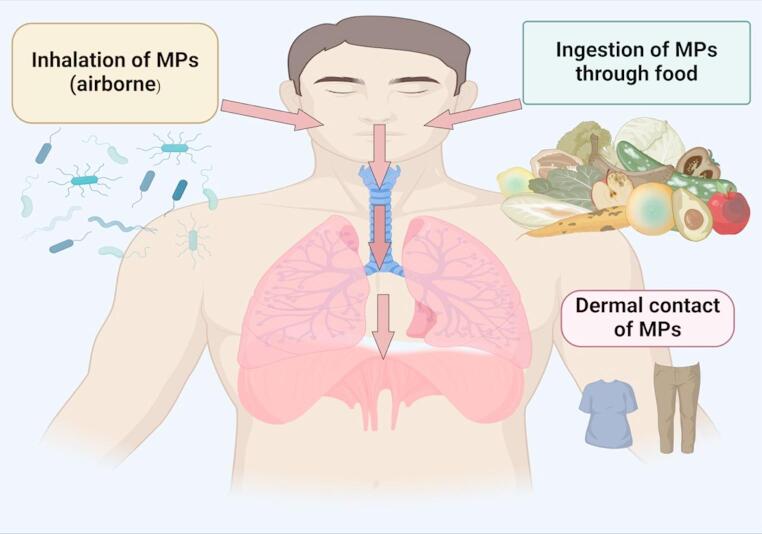
Routes of MP exposure and entry into the body

MPs occur in various forms, including plastic fragments, microbeads, paint particles, nurdles, films, tyre wear particles, and microfibres (Table 1) [5]. Human exposure to MPs primarily occurs through ingestion, especially *via* contaminated seafood, but also through beverages, inhalation of airborne particles (Table 2), and, to a lesser extent, dermal contact with products such as soaps, scrubs, or contaminated soil [5, 9, 10]. These multiple exposure pathways contribute to the accumulation of MPs in the human body, raising growing concerns about their potential health impacts (Fig. 1). Due to their synthetic composition and high molecular weight, MPs exhibit low biodegradability, allowing them to persist in both the environment and the human body [11], which may further exacerbate their long-term adverse effects.

In the plastic industry, the most commonly used plastics include low and high-density polyethylene (PE) (54.5%), polypropylene (PP) (16.5%), and polystyrene (PS) (9.7%) [12]. Mirroring this, in the aquatic environment, the most common types of MPs are polyethylene (PE) (25%), polyethylene terephthalate (PET) (16.5%), polypropylene (PP) (14%), and polystyrene (PS) (8.5%) [13]. From our review of the current literature, the most frequently used MP in research studies is PS. This is attributed to PS’s commercial availability, cost-effectiveness, surface chemistry (easily manipulated), and low variability (uniform density, charge, and hydrophobicity) [12, 14].

**Table 1:** A summary of different types of MPs, how these are formed, and the polymer type

Type of MPs	Formation Process for MPs	Polymer Type	Reference
Fragments	Degradation of larger plastics into MPs (~ 22% of marine MPs)	PE, PP, PS	[15, 16]
Paint particles	Shedding from painted surfaces or sandblasting (~ 58% of MPs, 1.9 million tonnes/year)	PU, PES, PS, polyacrylates, alkyls and epoxies	[17, 18]
Films	From agricultural mulch and thin flexible plastics (e.g., grocery or chip bags).	PE, PP, PS, PES, PET, PA, PU, PC	[19, 20]
Nurdles	Tiny particles are released during manufacturing, handling, and transport.	PE, PP, PS, PVC	[21, 22]
Microbeads	< 1 mm particles in personal care products (face wash, toothpaste).	PE, PET, PP, PA, PMMA	[23–25]
Tire wear particles	Abrasion from tires, releasing MPs with associated toxins, affects aquatic life.	PBR, SBR, PP, PE, PET	[26–28]
Microfibres	From textiles, clothing, fishing nets, ropes, cigarette filters, and other fibrous products.	PET, PA, PP, acrylic	[29–33]

Polyethylene (PE), Polypropylene (PP), Polystyrene (PS), Polyurethanes (PU), Polyamide (PA), Polyethylene terephthalate (PET), Polycarbonate (PC), Polyester (PES), Polyvinyl chloride (PVC), High-density Polyethylene (HDPE), Low-density Polyethylene (LDPE), Polyethylene vinyl acetate (PEVA), Polysulfone (PSU), Polymethyl Methacrylate (PMMA), Polybutadiene rubber (PBR), Styrene–butadiene rubber (SBR)

### MP characterisation

Plastics are typically characterized by particle size: mega-plastics, macro-plastics, meso-plastics, Microplastics, and Nano-plastics. MPs are defined as particles smaller than 5 mm but larger than 1 μm, and Nano-plastics (NPs) refer to particles smaller than 1 μm [10, 34, 35]. MPs can also be categorized based on their origin into primary and secondary types. Primary MPs are manufactured intentionally at small sizes and are commonly found in products like personal care items (e.g., microbeads in scrubs) and industrial abrasives. Examples include nurdles and microbeads [9, 10] (Table 1). In contrast, secondary MPs originate from the degradation and fragmentation of larger plastic items, such as packaging materials, textiles, or tyres. Environmental factors like UV radiation, mechanical abrasion, temperature, and humidity accelerate this fragmentation process [9, 10].

Furthermore, MPs are subject to environmental aging, which alters their physicochemical properties and may enhance their toxicity. A study on polystyrene MPs (PS-MPs) found that UV and weathered particles exhibited surface oxidation and structural damage, enhancing their ability to induce oxidative stress, immune activation, and neurodegenerative pathways, with weathered MPs showing stronger effects than UV-aged ones (Kim et al., 2023). Complementary in vitro tests on human microglial cells (HMC-3) further confirmed that weathered MPs triggered a more severe inflammatory response, underscoring the heightened neurotoxic potential of environmentally aged and weathered MPs.

**Table 2:** Summary of common sources, along with composition, size and concentration

	Details	Microplastic Type	Size and Concentration	References
Food				
Seafood	Marine ingestion of MP from polluted waters	PET, PP, HDPE, LDPE, PEVA, fiberglass	< 5 mm 0.02–1.60 particles/g of muscle tissue (depends on location and species)	[36–38]
Salt	MPs are due to ocean contamination	PE, PP, PET, PES	100–300 μm 33–313 particles/kg	[39]
Fruits and Vegetables	MPs due to soil contamination	PE, PP, PS	1.51–2.52 μm 52,600–307,750 μm/fruit 72,175–130,500 μm/vegetable	[40]
Dairy Products and Processed Foods	May be contaminated through animal feed, packaging, or processing	PES and PSU (Diary products) PET, PES, PP, PE (Processed Foods)	0.1 to 5 mm 6.5 ± 2.3 MPs/L (Diary products) 74 ± 220 particles/g of tissue (depends on processed food type)	[41, 42]
Drink				
Bottled Water	Plastic leaching from bottles and caps	PS, PET, PE	< 1 μm 2.4 ± 1.3 × 10^5^ particles/L	[43]
Tap Water	Depending on the water source and treatment infrastructure	PP, PE, PVC	32.4 μm 40 ± 16 MP/L	[44]
Soft Drinks	Plastic caps	PA, PET, PE	36–2228 μm 9.19 ± 1.84 MPs/L	[45]
Tea (plastic tea bags)	Release of MPs in hot water	Nylon, PET	20–100 nm 11.6 billion MPs/cup and 3.1 billion NPs/cup	[46]
Inhalation				
Indoor Air	MPs from synthetic textiles (carpets) and furniture	PET, PE, PP	4–398 μm 475 to 19,600 MPs/m^2^/day	[47]
Outdoor Air	MPs from tire wear, construction dust, and urban runoff	PET, PE, PP, PVC	0.004–3 mm 0.5 to 1357 MPs/m^2^/day	[47]
Textiles (clothing fibers)	Wearing and washing of synthetic clothing	PES, nylon	640,00–1,500,000 MPs/wash	[48]
Dust Inhalation	MPs from household dust	PE, PS, PP, PET	< 2 mm	[47]

### Health effects of MP exposure

Since the discovery of MPs in 2004, two decades of research have shown that these particles can adversely affect multiple human organ systems, including the digestive, respiratory, reproductive, and nervous systems (Table 3) [11, 49]. Moreover, MPs often carry toxic chemicals, which can leach into the body during digestion, exacerbating gastrointestinal symptoms [50]. Evidence also indicates that MPs can translocate from the gut into the bloodstream [51], allowing systemic distribution. Notably, MPs have been shown to cross the blood–brain barrier (BBB), enabling access to the central nervous system (CNS) [52, 53]. Within the CNS, MPs may induce oxidative stress, trigger inflammation, disrupt autophagy, impair mitochondrial function, interfere with acetylcholinesterase activity, and compromise the integrity of the BBB [49]. These disruptions can promote aberrant protein folding, neurotransmitter imbalances, and neuronal degeneration, leading to both cognitive and physiological impairments [49, 52]. Such changes are increasingly linked to the onset and progression of neurodegenerative diseases, including Alzheimer’s disease (AD) and Parkinson’s disease [1, 49, 54]. Despite growing concern and accumulating evidence, a comprehensive understanding of the molecular and cellular mechanisms by which MPs contribute to the development and progression of neurodegenerative diseases remains limited. This review aims to synthesise emerging data on the link between MPs and neurodegenerative diseases, with a particular focus on AD and PD, the two most prevalent diseases in this category [55]. By doing so, it seeks to clarify potential pathogenic pathways and stimulate further research into the neurological impacts of microplastic exposure.

**Table 3:** A comparison of different types of MPs and their potential health effect

Plastics	Potential health effects of plastic	References
Mega-plastics (> 100 mm)	Fragment into MPs; ingested or inhaled.	[34]
Macro-plastics (20 mm to 100 mm)	Break down into MPs; ingested or inhaled.	[34]
Meso-plastics (5 mm to 20 mm)	It can be ingested, causing irritation and inflammation in the stomach, leading to gastrointestinal symptoms such as abdominal pain, nausea, and vomiting.	[11]
Microplastics (1 μm to 5 mm)	It can either be inhaled or ingested, causing adverse health outcomes. These include: • Gastrointestinal symptoms – abdominal pain, nausea and vomiting. • Respiratory symptoms – shortness of breath, sneezing and coughing. • Endocrine effects and disorders – developmental, reproductive (i.e., miscarriage, congenital malformations, and infertility), and metabolic. • Neurological problems – oxidative stress, inflammatory responses, mitochondrial dysfunction, disorganized acetylcholinesterase activity, and impaired autophagy. • Cardiovascular problems – increased risk of heart attack and stroke.	[11, 49, 52, 56]
Nano-plastics (< 1 μm)	Inhaled or ingested like MPs; more toxic due to smaller size; may cause DNA damage, tumor formation, and act as carcinogens in high-renewal tissues.	[11, 43, 56, 57]

## Neurodegenerative diseases

Neurodegenerative diseases are typically marked by progressive loss of neurons in the CNS [55]. The most prevalent conditions in this category are AD and PD, though others include Amyotrophic Lateral Sclerosis (ALS), Huntington’s disease, prion diseases, motor neuron diseases, spinocerebellar ataxia, and spinal muscular atrophy. Neurological disorders affect more than one-third of the global population and are among the leading causes of disability and illness globally [2, 58]. This review focuses on AD and PD due to their high prevalence and public health impact.

### Alzheimer’s disease and epidemiology

AD is a neurodegenerative disorder primarily defined by the accumulation of amyloid-beta (Aβ) plaques and hyperphosphorylated tau proteins, the key pathological features characteristic of the disease [55, 59]. Research suggests that MPs crossing the BBB may disrupt protein folding, promote the production of abnormal amyloid proteins, and contribute to amyloidosis, hallmarks of AD pathology [49].

 [60, 61].

Globally, over 57 million people live with dementia, with AD accounting for 60–70% of cases; this is projected to rise to 152 million by 2050 [62, 63]. In the U.S., nearly 7 million individuals are affected, making AD the fifth leading cause of death in those aged ≥ 65 in 2021 [64]. Prevalence increases sharply with age, with a 15-fold higher risk between 60 and 85 years [65]. Women face nearly double the risk of men, with a 1 in 5 lifetime risk at age 45 compared to 1 in 10 for men [64]. The economic burden is substantial, with care costs projected to reach $360 billion in 2024 and nearly $1 trillion by 2050.

#### AD pathogenesis and pathophysiology

The primary cause of AD remains unclear, but hallmark features include extracellular Aβ plaques and intracellular tau-based neurofibrillary tangles (NFTs) [66, 67]. Normally, amyloid precursor protein (APP) is cleaved by α- and γ-secretases to form soluble peptides, but in AD, β- and γ-secretases generate insoluble Aβ fibrils that aggregate into plaques, disrupting synaptic signaling and promoting neurodegeneration [68–71].

Aβ aggregation triggers tau hyperphosphorylation, leading to NFTs that destabilize microtubules and impair neuronal transport [72, 73]. Both Aβ and tau disrupt mitochondrial dynamics, causing energy deficits, oxidative stress, and cell death [74]. Impaired autophagy-lysosomal pathways further prevent clearance of misfolded proteins, worsening their accumulation [75].

Chronic neuroinflammation is another hallmark, with activated microglia and astrocytes releasing cytokines that amplify neuronal damage [76, 77]. Amyloid angiopathy reduces cerebral blood flow and increases hemorrhage risk [78]. These processes compromise blood–brain barrier integrity, fueling a cycle of oxidative stress and immune infiltration [79, 80].

Pathological progression typically begins with Aβ plaques in the neocortex spreading to deeper brain regions, while NFTs emerge in the locus coeruleus and transentorhinal regions before advancing [71].

### Parkinson’s disease and epidemiology

PD is a progressive neurodegenerative disorder linked to environmental exposures, including MPs [81]. It is primarily characterized by the degeneration of dopaminergic neurons in the substantia nigra, leading to dopamine deficiency in the striatum and the accumulation of intracellular protein aggregates known as Lewy bodies, which contain misfolded α-synuclein [82]. Emerging research suggests that MPs may contribute to the formation of these abnormal α-synuclein fibrils, thereby playing a potential role in PD pathogenesis [81].

 [83].

Globally, more than 10 million people live with PD, which primarily affects those over 65, with incidence rising sharply with age [84]. In the U.S., PD is the second most common neurodegenerative disorder after AD, with annual incidence rates of 108–212 per 100,000 in people over 65 and 47–77 per 100,000 in those over 45 [85]. PD caused 329,000 deaths in 2019—a > 100% increase since 2000 [83]. It is more common in men, who are ~ 1.5 times more likely to develop it [84], affecting 1–2 per 1,000 and ~ 1% of people over 60 [86]. The economic burden is significant, with U.S. costs estimated at $52 billion annually, and prevalence influenced by socioeconomic disparities [87].

#### PD pathogenesis and pathophysiology

**Fig. 2:**
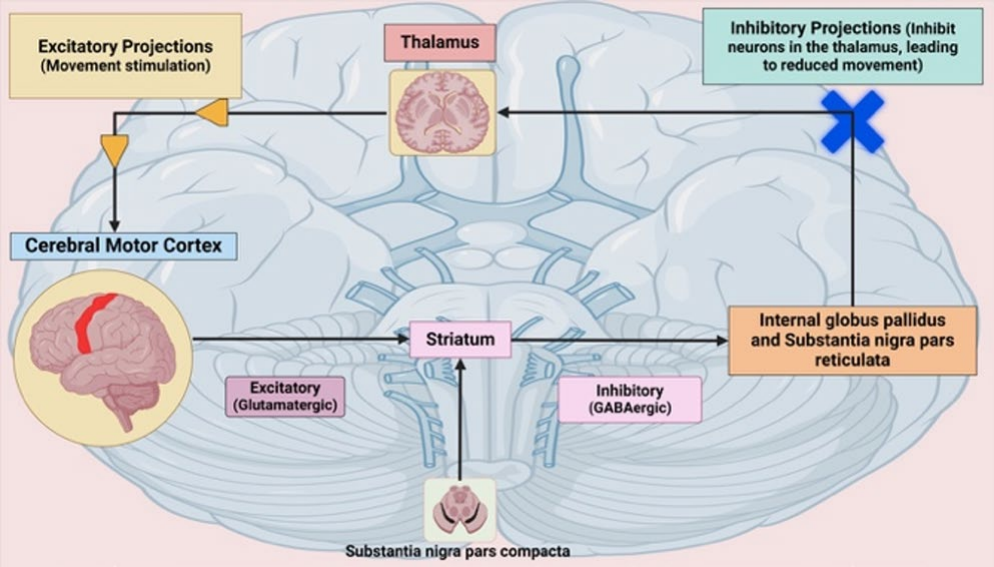
Simplified flowchart of Direct Pathway

**Fig. 3:**
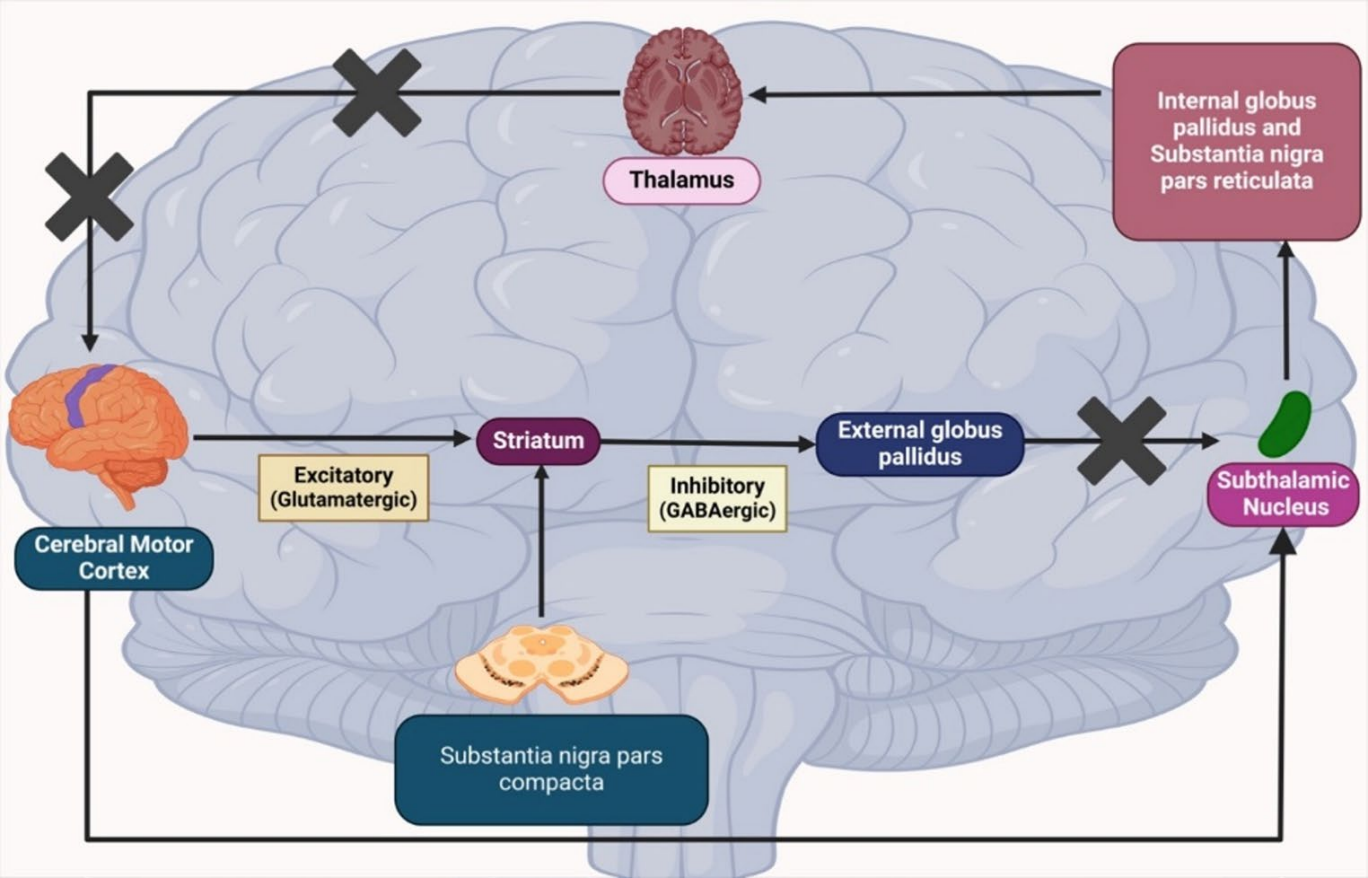
Simplified flowchart of the Indirect Pathway

Like AD, the exact cause of PD is unclear, but key mechanisms include mitochondrial dysfunction, oxidative stress, α-synuclein misfolding, and chronic neuroinflammation [82, 88]. Motor control depends on three pathways: direct, indirect, and hyper-direct. Dopamine enhances the direct pathway via D1 receptors, facilitating movement, and inhibits the indirect pathway via D2 receptors, reducing motor suppression, thereby coordinating voluntary movement [89–92]. The pathophysiology of PD is shown in Fig. 2 and 3.

In PD, degeneration of dopaminergic neurons in the substantia nigra pars compacta causes striatal dopamine deficits, disrupting the balance between direct and indirect pathways and producing hallmark symptoms such as tremor, bradykinesia, rigidity, and postural instability [92]. Neuronal loss is multifactorial, with genetic mutations, environmental toxins, mitochondrial dysfunction, and neuroinflammation implicated [93]. A pathological hallmark is Lewy bodies, intracellular inclusions primarily composed of misfolded α-synuclein, which are thought to impair neuronal function and contribute to motor, cognitive, and behavioural deficits [94, 95] [96].

## Microplastic-induced blood–brain barrier dysfunction

As previously discussed, MPs are capable of crossing the BBB, triggering a cascade of molecular and cellular responses that can harm brain cells and tissues. This includes disruption of reactive oxygen species (ROS) homeostasis, leading to oxidative stress, as well as mitochondrial dysfunction, impaired autophagy, neuroinflammation, and interference with neurotransmitter systems such as acetylcholinesterase activity [49, 52]. These pathological changes can result in protein misfolding, neuronal loss, and neurotransmitter imbalances, ultimately manifesting as abnormal behaviours and significant physiological and cognitive impairments. Collectively, these effects contribute to the development and progression of neurodegenerative diseases such as AD and PD [49, 52].

### MP’s ability to cross the BBB

Since the detection of MPs in brain tissue, an important question has emerged: how do MPs cross the BBB [52, 97]. The ability of microparticles to traverse biological barriers such as the BBB and intestinal mucosa depends on several factors, including particle size, surface charge, surface chemistry, and the types of cells they encounter [97]. Using an engineered model, Cho et al. examined the toxicity and size-dependent absorption of polystyrene microplastics at the BBB. The findings demonstrated that 0.2 μm particles caused bigger increases in permeability (15.6-fold vs. 2-fold at 24 h; 27.3-fold vs. 4.5-fold at 72 h) due to their significantly higher uptake and transendothelial transport compared to 1.0 μm particles. BBB degradation and microplastic absorption were further enhanced by exposure in conjunction with TNF-α. Crucially, toxicity varied between 3D BBB models and 2D cell cultures, highlighting the necessity of sophisticated models for evaluating microplastic neurotoxicity {Cho, Yeongseon, 2024}.

For particles larger than 0.5 μm, translocation across the BBB is thought to occur via receptor-mediated phagocytosis, where MPs bind to cell surface receptors, triggering vesicle formation and fusion with lysosomes [97]. These larger MPs may also be taken up through endocytosis, in which BBB cells engulf the particles and transport them across the barrier. In contrast, smaller MPs (less than 0.5 μm) are believed to cross cellular membranes through transcytosis, diffusing across the lipid bilayer and exiting the cell on the opposite side without being fully internalised [98]. NPs, due to their extremely small size and high surface area-to-volume ratio, can directly penetrate biological membranes, potentially causing structural and functional cellular damage [57].

**Fig. 4:**
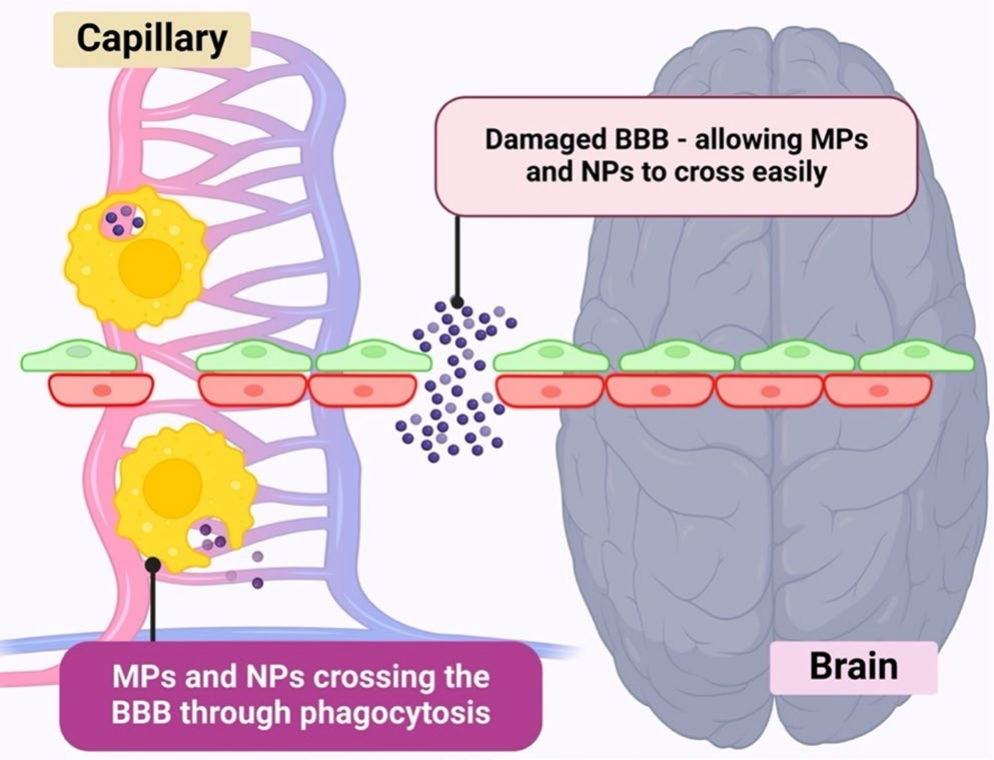
MP’s ability to cross the BBB through phagocytosis and BBB damage

Additionally, conditions such as stroke and traumatic brain injuries (e.g., concussion) can compromise the integrity of the BBB [99]. Similarly, ageing can alter key structural components of the BBB, weakening its function and overall integrity [100]. Under these circumstances, MPs and NPs are more likely to cross the BBB, accelerating their accumulation within the brain [101] (Fig. 4). Thus, in both healthy and compromised states, MPs and NPs are capable of breaching the BBB through various mechanisms, potentially initiating neurotoxic effects within brain tissue (Fig. 4).

Interestingly, an animal model study demonstrated that orally administered polystyrene microplastics (PS-MPs) were able to cross the BBB and reach the brain within just two hours of ingestion [97]. To further investigate the underlying mechanism, the researchers employed coarse-grained molecular dynamics simulations. These simulations revealed the formation of a surface structure known as the *biomolecular corona*, which plays a crucial role in facilitating the passive transport of MPs across the BBB. Furthermore, the presence of cholesterol molecules was found to enhance the integration of MPs into the BBB membrane, further promoting their uptake [97]. These findings suggest that the biomolecular corona is a key factor enabling the passive translocation of MPs into the brain. For another potential route facilitating MP brain entry, a recent study conducted in 2024 examined the olfactory bulbs of 15 deceased individuals during routine coroner autopsies and detected the presence of MPs in 8 of the cases [102]. Based on these findings, the researchers proposed that inhaled MPs can traverse the nasal cavity and reach the olfactory bulb. This pathway is of particular significance because it bypasses the BBB, thereby providing a direct route for MPs to access brain tissue. Consequently, the olfactory route may represent a critical mechanism by which inhaled MPs enter the brain.

### MP’s distribution within the brain

Although MPs can cross the BBB, their distribution and accumulation within the brain remain largely unknown. A 2024 study by Campen et al. compared MP accumulation in human kidneys, livers, and brains using tissue samples collected between 2016 and 2024. Their analysis revealed that brain tissues had the highest MP concentrations, with levels in all organs increasing over time. Notably, because all brain samples were taken from the frontal cortex, a region critical for cognitive functions, this suggests that MPs may preferentially accumulate in this area [103]. However, no comparable data are yet available for other brain regions such as the hippocampus, cerebellum, or basal ganglia, leaving open the question of whether MP accumulation shows region-specific patterns. Expanding on this work, the same research group published a 2025 study [104], which reported significant MP accumulation in immune cells, such as glial cells, and cerebrovascular walls in individuals with documented dementia. This suggests that MPs may localize in inflammatory and vascular regions of the brain, particularly in disease states. Together, these findings suggest that MPs may not be uniformly distributed but instead concentrate in regions critical for cognition (frontal cortex) or associated with vascular and inflammatory activity, while other brain regions remain insufficiently characterized. While these findings provide early insights into MP distribution, the mechanisms by which MPs migrate within the brain remain poorly understood.

Based on current literature, three potential mechanisms by which MPs may migrate throughout the brain are proposed: movement via cerebrospinal fluid (CSF), transport through the neurovascular system, and uptake by glial cells. The detection of MPs in CSF [101] suggests that this fluid may serve as a medium through which MPs are distributed across different brain regions. Further, the presence of MPs in immune cells and cerebrovascular walls [104] implies that MPs may circulate through the vascular system or be taken up by immune-related glial cells, enabling their transport within the neurovascular network. Despite these emerging insights, further research is needed to map MP accumulation across brain regions comprehensively and to clarify the mechanisms governing their internal distribution. Future research should prioritize systematic comparisons of MP accumulation across distinct brain regions to determine whether certain areas are more vulnerable, thereby linking exposure patterns to specific neurological functions.

## Microplastics and possible shared mechanisms between alzheimer’s and parkinson’s

This section seeks to examine the shared mechanisms underlying the interactions between MPs, AD, and PD. However, much of the current evidence is derived from in vitro and animal studies, with limited data available from human clinical research, which poses challenges for direct translation to human pathophysiology. Consequently, these findings should be interpreted with caution due to the paucity of human evidence. Nevertheless, elucidating and highlighting the potential mechanistic links between MPs, AD, and PD may provide valuable insights and open novel avenues for future therapeutic development.

### Cellular homeostasis disruption

#### Neuroinflammation

Considering the ability of MPs to penetrate the BBB and disperse throughout the brain, a primary concern is their potential to initiate neuroinflammatory responses [52]. This idea is compounded by the idea that the toxicity of MPs and NPs increases as the time in the brain increases compared to newer particles, as a result of particle aggregation and adsorption of bioactive compounds [105].

Based on existing data regarding MP distribution and accumulation in the brain, the specific regions where MPs build up may modulate their impact on brain function, potentially contributing to different neurodegenerative processes and the development of neurodegenerative diseases [102, 103]. For example, if MPs were to be distributed within the frontal cortex and left for a prolonged period, it could lead to neuroinflammation and the development of accumulation of Aβ, thus leading to AD [103]. In contrast, if MPs were to accumulate within dopaminergic neurons, such as that seen in Prüst’s study, it could lead to PD [105].

In terms of neuroinflammation, many studies have shown an association between MPs and neuroinflammation. From our literature search, our review identified five main mechanisms through which MPs can cause neuroinflammation: activation of native immune cells within the brain (e.g., microglia and astrocytes), oxidative stress, disruption of the BBB, mitochondrial dysfunction, and damage to neural structures. These mechanisms work in tandem to ultimately increase neuroinflammation within the brain.

Among other things, high metal levels lead to oxidative stress, misfolded proteins, mitochondrial dysfunction, dysregulated autophagy, and apoptosis. One study investigated how heavy metals, like as copper (Cu), arsenic (As), cadmium (Cd), iron (Fe), and lithium (Li), affect the pathological circumstances of Parkinson’s disease [1] and how they relate to neurodegeneration [106]. Heavy metals can attach covalently to the surfaces of microplastics and enter biological systems, where they can cause neuronal damage, mitochondrial malfunction, and oxidative stress. According to different research, a compromised metal homeostasis caused by increasing metal exposure could be a factor in neurological diseases [107]. Persistent organic pollutants, which can build up in neurons, interfere with endocrine signaling and worsen inflammation-related neurotoxicity, and are transported by microplastics. Polycyclic aromatic hydrocarbons, which are frequently linked to microplastic surfaces, can penetrate the blood–brain barrier, harm DNA, and disrupt synaptic function [9, 108]. Compounds from pesticide residues can be consumed and bioaccumulate when attached to microplastics, disrupting neurotransmission and making neurons more vulnerable [105].

#### Activation of native immune cells

MPs are recognized as foreign entities within the body, which triggers their phagocytosis by microglia, thereby activating these cells [109]. For instance, a 2022 study demonstrated that PS-MPs were phagocytosed by microglia, leading to polarization of the cells. This polarization resulted in a hypertrophied morphology and upregulation of both M1 and M2 microglial markers, ultimately culminating in microglial apoptosis [110]. Likewise, a 2024 in vitro study showed that polystyrene nanoplastics (PS-NPs) were primarily taken up by microglia, triggering activation, morphological changes, and neuroinflammatory gene expression that may contribute to cognitive deficits [109].

**Fig. 5:**
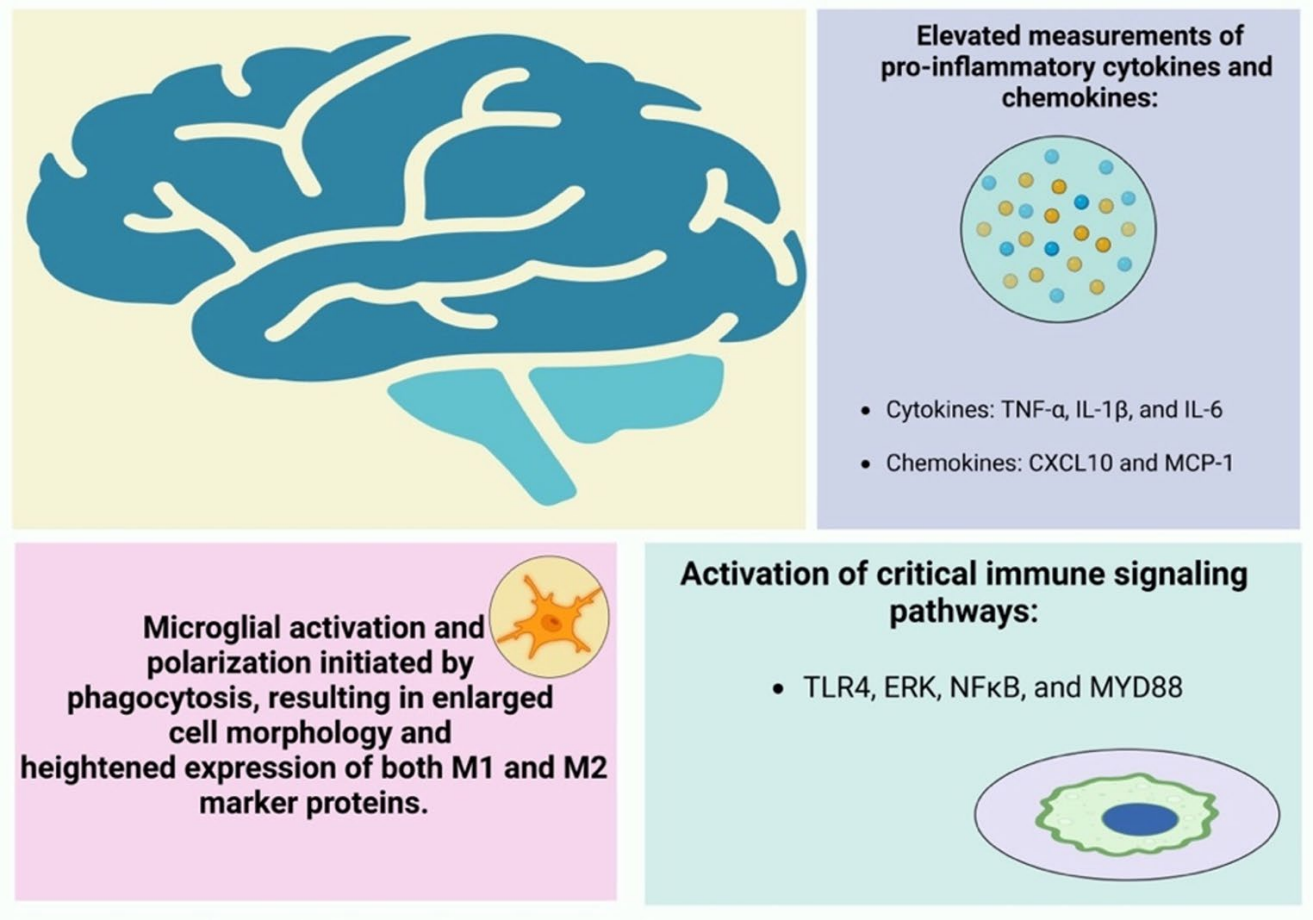
MP induced neuroinflammation by microglial phagocytosis, elevated pro-inflammatory cytokines and chemokines, and activation of critical immune signalling pathways

Furthermore, microglial activation was associated with elevated levels of pro-inflammatory cytokines, including TNF-α, IL-1β, and IL-6, as well as chemokines such as CXCL10 and MCP-1 [110, 111]. These findings suggest that MPs play a significant role in inducing neuroinflammation. The activation of key immune signaling pathways, evidenced by increased protein concentrations and phosphorylation of TLR4 (Toll-like receptor 4), ERK, NFκB, and MYD88 following MP exposure in mice, further substantiates this hypothesis [111–113] (Fig. 5).

Beyond microglia, MPs also activate astrocytes. A three-week study on PS-MP-exposed mice assessed the presence of glial fibrillary acidic protein (GFAP) as an indicator of astrocyte activation [52]. GFAP, a critical intermediate filament protein in mature astrocytes, plays a vital role in processes such as autophagy, neurotransmitter uptake, and astrocyte development [114]. As such, GFAP serves as a marker of neuroinflammation and astrocyte activation. Interestingly, the study found a slight decrease in GFAP expression in older mice and a more significant decline in younger mice following MP exposure [52]. While this response deviated from the typical inflammatory pattern, it aligned with previous studies suggesting that GFAP expression may decrease in the early stages of neurodegenerative diseases like Alzheimer’s disease [115, 116]. These findings imply that astrocyte atrophy and reduced GFAP expression may be early markers of disease onset, contributing to the pathogenesis of neurodegenerative conditions [52].

#### Oxidative stress

A 2024 review by Kadac-Czapska et al. explored the notion that MPs induce oxidative stress primarily through two mechanisms: increasing ROS production and impairing the body’s antioxidant defense systems. Together, these effects disrupt cellular redox homeostasis—the balance between ROS generation and antioxidant activity, thereby promoting oxidative stress [117].

MPs elevate ROS levels via both intracellular and extracellular pathways. Intracellularly, MPs can cross biological barriers such as the BBB through phagocytosis, endocytosis, or transcytosis. Once internalized, MPs may localize to mitochondria via biofilm-mediated transport, leading to mitochondrial membrane potential disruption and excessive ROS production. Additionally, when internalized by immune cells like microglia, MPs stimulate inflammatory responses and ROS release [117].

Extracellularly, environmental degradation processes, such as ultraviolet radiation, heat, and humidity, fragment MPs, facilitating the formation of free radicals on their surfaces. These radicals arise through mechanisms such as hydrogen abstraction from polymer chains or addition to unsaturated bonds. Upon contact with atmospheric oxygen, these radicals form secondary ROS, including superoxide and alkyl radicals, which can propagate oxidative damage [117].

Furthermore, MPs impair the body’s antioxidant capacity by reducing the activity of key enzymes such as superoxide dismutase (SOD), catalase (CAT), and glutathione S-transferase (GST). For instance, a 2024 mouse model study by Kehinde et al. demonstrated that exposure to polyethylene microplastics (PE-MPs) led to significant reductions in GST, CAT, SOD, glutathione (GSH), and ascorbic acid levels. Concurrently, levels of malondialdehyde (MDA)—a biomarker of lipid peroxidation and oxidative stress—were elevated, confirming MP-induced oxidative damage [118]. This idea was supported by two separate studies, a 2022 study on Kunming mice exposed to PS-MPs, where exposure increased ROS and MDA concentrations, while decreasing glutathione, and another 2023 study on Wistar rats exposed to MPs significantly decreased SOD expression but increased neuronal damage (shown through the expression of MDA) and neuronal deoxyribonucleic acid damage [119]. Overall, this oxidative stress is also able to cause the further activation of microglia and astrocytes, amplifying neuroinflammation within the brain [120].

#### Disruption of the blood-brain barrier

A recent 2025 study by Kim et al. demonstrated that MPs could cross the BBB and downregulate the expression of key tight junction proteins, including claudins, occludin, and zonula occludens-1 (ZO-1). This downregulation disrupts the structural integrity of the BBB, leading to increased permeability [121]. Additionally, the subsequent activation of immune cells within the brain triggers the release of pro-inflammatory cytokines, such as TNF-α, IL-1β, and IL-6, which further damage BBB endothelial cells [122]. This inflammatory response also promotes the activation of matrix metalloproteinases (MMPs), enzymes that degrade the extracellular matrix and tight junction components, exacerbating BBB disruption [123]. Whether through increasing BBB permeability or directly impairing its function, MPs ultimately compromise the barrier, allowing the infiltration of peripheral immune cells (e.g., neutrophils) and other harmful agents, including additional MPs, into the brain, thereby contributing to neuroinflammation [124].

#### Mitochondrial dysfunction

Mentioned before, when MPs are endocytosed into cells, they are able to translocate to the mitochondria, increasing ROS. However, MPs are also able to impair mitochondria function by depolarising the mitochondrial membrane [125]. Microplastics disrupt oxidative phosphorylation in mitochondria, leading to impaired adenosine triphosphate (ATP) production, which in turn compromises energy-dependent neuronal functions and contributes to neuronal damage [126]. These damaged neurons release damage-associated molecular patterns (DAMPs), further activating immune responses and therefore causing neuroinflammation.

#### Damage to neural structures

MPs have been shown to physically damage various neural structures, including the myelin sheath, microtubules, and neuronal membranes [127]. An example of this is MPs’ damage to myelin sheath, seen through a study where NPs had reduced cerebellar medullary sheath thickness and related protein expression (e.g., myelin basic protein and myelin oligodendrocyte glycoprotein) [128]. As a result of this structural injury in neural structures, this can activate pattern recognition receptors (PRRs), such as TLRs, on immune cells, triggering neuroinflammation [129].

## Potential mechanisms for MPs specific to alzheimer’s disease

**Fig. 6:**
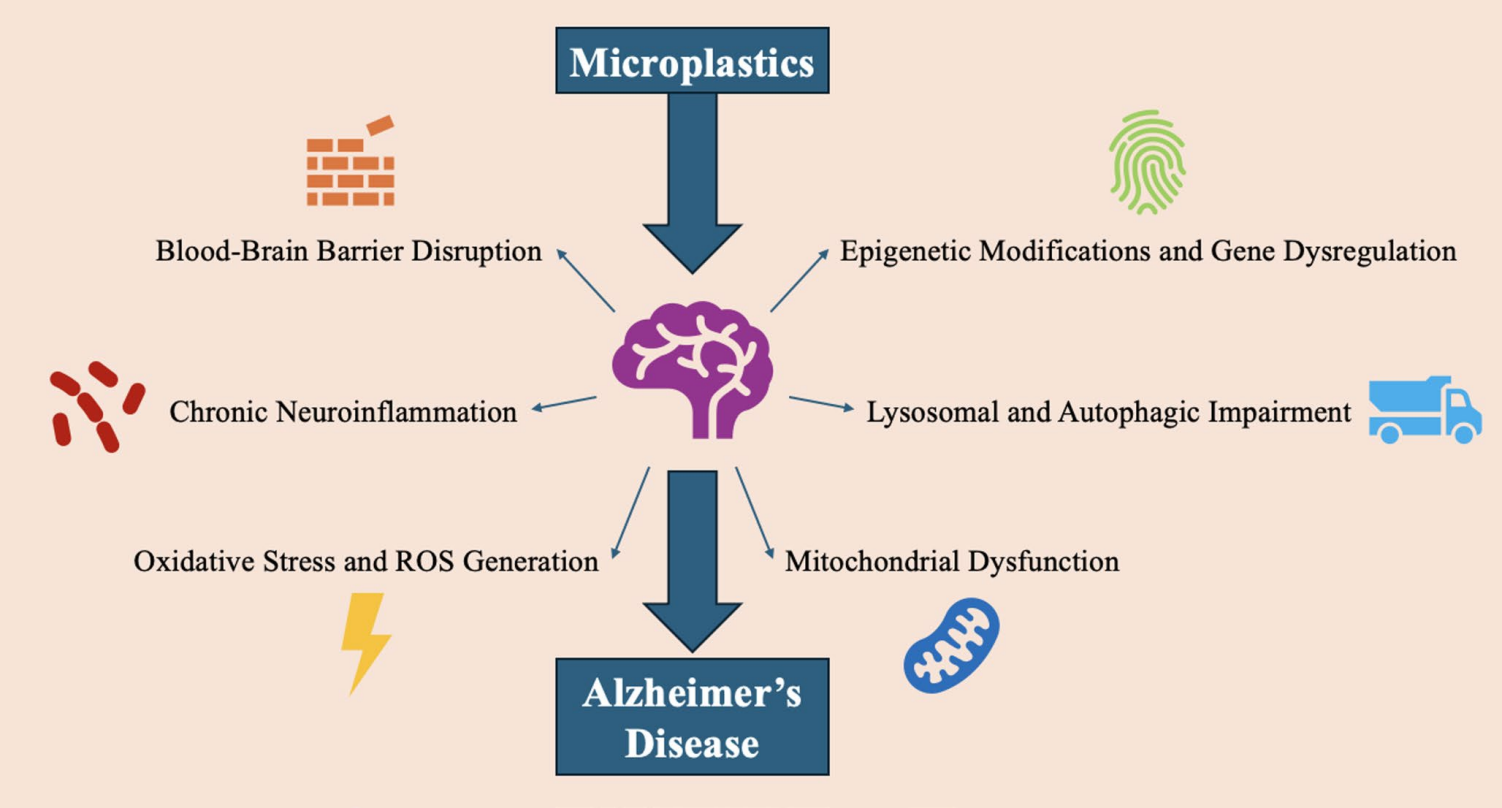
Potential Mechanisms for MPs in the development and progression of Alzheimer’s Disease

In recent years, MPs have been inextricably linked with the development and progression of AD. From our review of the current literature, this review found six potential mechanisms by which MPs are able to contribute to AD. These include BBB disruption, chronic inflammation, oxidative stress and ROS generation, mitochondrial dysfunction, autophagy and proteostasis impairment, and epigenetic modifications and gene dysregulation (Fig. 6). As some of the potential mechanisms have already been discussed, this section will go into more detail on other potential mechanisms, more specific to AD.

### Blood-brain barrier disruption

MPs have been shown to cross the BBB and significantly disrupt its function by downregulating key junction proteins such as claudins, occludin, and ZO-1, thereby compromising its structural integrity. For example, a 2022 study found that exposure to MPs in an in vitro BBB model reduced the expression and release of junction proteins, including occludin and zonulin [112]. Similarly, two independent in vivo studies reported that MP exposure increased BBB permeability in the hippocampus, hypothalamus, and cortex of mice [130, 131].

Notably, early BBB breakdown and dysfunction play a critical role in the development and progression of AD [132]. Given this, it shows that MPs may contribute to AD progression by disrupting the BBB. A more permeable BBB could allow increased infiltration of neurotoxic substances, including MPs themselves, cytokines, and immune cells such as neutrophils [133]. This, in turn, may exacerbate neuroinflammation, a key driver of AD pathogenesis [134, 135].

### Chronic neuroinflammation

MPs can persist and accumulate in brain tissue, with post-mortem analysis showing a 50% increase in concentrations between 2016 and 2024 [104]. Their chronic presence may sustain neuroinflammation by repeatedly activating immune pathways. MPs stimulate microglia and astrocytes to release cytokines such as TNF-α, IL-1β, and IL-6 [110], which recruit neutrophils across a compromised BBB [124]. Neutrophils contribute to AD-like pathology through infiltration, NET formation, and amplification of cytokine release, reinforcing a cycle of sustained inflammation [136–139].

Chronic neuroinflammation promotes Aβ deposition and tau hyperphosphorylation. Early studies linked inflammation to Aβ accumulation [140], and more recent work showed a biphasic inflammatory response: rising with early Aβ, declining with heavy Aβ load, and re-emerging with tau accumulation [141]. MPs may also activate the NLRP3 inflammasome through ROS generation, lysosomal damage, and DAMP release [142]. Since NLRP3 is also activated by Aβ and tau [143], this pathway amplifies IL-1β and IL-18 release, driving neurodegeneration and cognitive decline.

### Oxidative stress and ROS generation

Earlier in this review, a connection between MPs, ROS, oxidative stress, and neuroinflammation had been established. Building on this, MP-induced oxidative stress may contribute to the onset and progression of AD. This idea is supported by evidence showing that oxidative stress occurs early in AD pathogenesis, often preceding the formation of amyloid plaques and neurofibrillary tangles [144]. Given that MPs can promote ROS production, chronic MP exposure may sustain oxidative stress in the brain, potentially accelerating AD development.

A 2022 study using Kunming mice further supports this theory, linking PS-MP exposure with increased ROS levels and neuronal disruption in the hippocampus, including a reduction in Nissl bodies [145]. Since the hippocampus is one of the first regions affected in AD and is essential for memory and spatial navigation [146], these findings suggest that MPs may contribute to AD-related neurodegeneration.

In addition, MPs have been shown to interact with misfolded proteins involved in AD pathology. A 2024 study reported that mice exposed to 100 pM PS-NPs had significantly increased expression of Aβ40 and Aβ42 due to enhanced nucleation, promoting amyloid-beta oligomer formation and neurotoxicity [59]. The hydrophobic nature of polystyrene nanoparticles likely facilitates binding between Aβ monomers, increasing aggregation and toxicity. This mechanism suggests MPs may trigger both systemic and localized amyloidosis [147], contributing to AD development.

Moreover, oxidative stress can impair proteolytic enzymes responsible for clearing Aβ, including insulin-degrading enzyme, endothelin-converting enzymes 1 and 2, and neprilysin [148]. Inhibition of these enzymes by oxidative stress results in Aβ accumulation, further exacerbating AD pathology.

### Mitochondrial dysfunction

As previously discussed, MPs once internalized can localize to mitochondria where they disrupt mitochondrial membranes, elevate ROS production, and impair ATP synthesis, ultimately leading to mitochondrial dysfunction [125]. In addition, MPs physically damage mitochondrial structures and reduce the organelle’s energy-generating capacity.

Mitochondrial dysfunction can have several downstream effects, including neuronal energy deficits, activation of apoptosis, and the exacerbation of tau-related pathologies. Reduced ATP production, due to impaired function of electron transport chain enzymes, can lead to an insufficient energy supply in neurons [149]. Disruption of mitochondrial membrane potential causes calcium ion (Ca²⁺) imbalance, which further impairs ATP synthesis and activates key apoptotic enzymes such as caspase-9 and caspase-3, triggering the mitochondrial apoptosis pathway [150].

In terms of tau pathology, decreased ATP levels impair axonal transport, which is essential for moving organelles and proteins, including tau, along the neuron [151]. This disruption can lead to the accumulation and misfolding of tau within neurons, compromising microtubule stability due to tau’s critical structural role [152]. Consequently, this promotes the formation of toxic tau aggregates.

These processes are highly relevant to AD, in which mitochondrial dysfunction and tau aggregation are hallmark features [74]. Therefore, MPs contribute to AD development and progression by inducing mitochondrial dysfunction, triggering neuronal energy failure and apoptosis, and promoting tau aggregation.

### Lysosomal and autophagic impairment

MPs have also been shown to interfere with both lysosomal and autophagic pathways. Regarding lysosomal function, MPs can exert both direct and indirect effects [117]. A direct impact occurs when cells internalize MPs through endocytosis. In attempting to digest these particles, the lysosomes become disrupted, leading to impaired function [153, 154]. Indirectly, MPs elevate oxidative stress, which destabilizes lysosomal membranes due to their sensitivity to reactive oxygen species (ROS), ultimately resulting in lysosomal dysfunction [155].

In terms of autophagic disruption, a 2022 study demonstrated that PS-MPs triggered autophagy-dependent ferroptosis and apoptosis in the cerebellum of chickens by inhibiting the Nrf2/Keap1/HO-1/NQO1 signaling pathway [156]. This finding was supported by a more recent study using immortalized mouse brain endothelial cells, where PS-NPs inhibited autophagy, leading to elevated intracellular iron levels. This inhibition was associated with increased BBB permeability and damage to tight junction proteins [121].

The lysosomal and autophagic pathways play essential roles in maintaining brain homeostasis by clearing cellular waste and degrading damaged components, including misfolded proteins [157]. The evidence points to the idea that MPs disrupt these clearance mechanisms, resulting in the accumulation of pathological proteins such as Aβ plaques and neurofibrillary tangles. This disruption could therefore contribute to the development and progression of AD. Notably, impaired lysosomal and autophagic function is a well-established feature of AD pathology [158], further supporting the potential link between MP-induced pathway disruption and AD pathogenesis.

### Epigenetic modifications and gene dysregulation

MPs have been increasingly implicated in inducing epigenetic modifications within animal cells [159, 160]. A 2022 study demonstrated that zebrafish embryos exposed to MPs exhibited significant downregulation of genes involved in neuronal proliferation (*sox2*, *pcna*), neurogenesis (*neuroD*, *olig2*), and motor neuron development (*islet*) [161]. Additionally, researchers observed reduced expression of both maintenance and de novo DNA methyltransferases, highlighting MPs’ influence on DNA methylation and suggesting a potential mechanism through which MPs may impair neurogenesis.

Similar findings were reported in another 2022 study, where zebrafish embryos exposed to PS-MPs displayed DNA hypomethylation following repeated exposure. This was accompanied by increased expression of oxidative stress-related genes (*sod2* and *nrf2a*) [162]. In adult zebrafish, PS-MP exposure led to upregulation of genes such as *gstp1* (antioxidant), *hsp70l* (heat shock protein), and *ptgs2a* (inflammatory marker), alongside downregulation of key antioxidant and neuronal genes including *cat*, *sod1*, *gpx1a*, and *ache* [163].

Genes such as *sod1*, *cat*, and *gpx1a* are essential for neutralising ROS and maintaining brain homeostasis [164]. These same genes are also known to be downregulated in AD [165, 166]. Thus, postulate that MP-induced epigenetic modifications, particularly DNA methylation changes affecting these genes, may contribute to AD pathogenesis by increasing ROS levels and promoting oxidative stress, a central feature of the disease.

Moreover, MPs have been shown to influence neuronal microRNAs (miRNAs). For instance, a 2021 study found that exposure to PS-NPs significantly downregulated *mir-76*, a miRNA involved in regulating heme homeostasis and providing neuroprotection [167]. This downregulation led to elevated *glb-10* expression and increased ROS production, supporting our hypothesis that MP-induced epigenetic changes may contribute to neurodegenerative processes. Importantly, oxidative stress has also been shown to influence epigenetic mechanisms [168], suggesting a possible feedback loop wherein epigenetic changes enhance oxidative stress, which in turn reinforces further epigenetic disruption. Finally, a recent review identified several human miRNA pathways potentially affected by MP exposure, including KSR-ERK-MAPK, FOXO-Insulin, and GPX3-HIF-α [169]. These pathways regulate critical cellular processes such as glucose metabolism, apoptosis, cell proliferation, angiogenesis, and protein folding—all of which are dysregulated in AD. Collectively, these findings reinforce the idea that MP-induced epigenetic alterations could play a role in exacerbating AD pathogenesis and/or progression. However, currently, much of this research is within animal or cell models, especially with high levels of MP exposure, making it difficult to extrapolate these findings. However, nevertheless, these models provide critical mechanistic insights and highlight potential pathways MPs may contribute to ADs.

## Potential mechanisms for MPs specific to parkinson’s disease

**Fig. 7:**
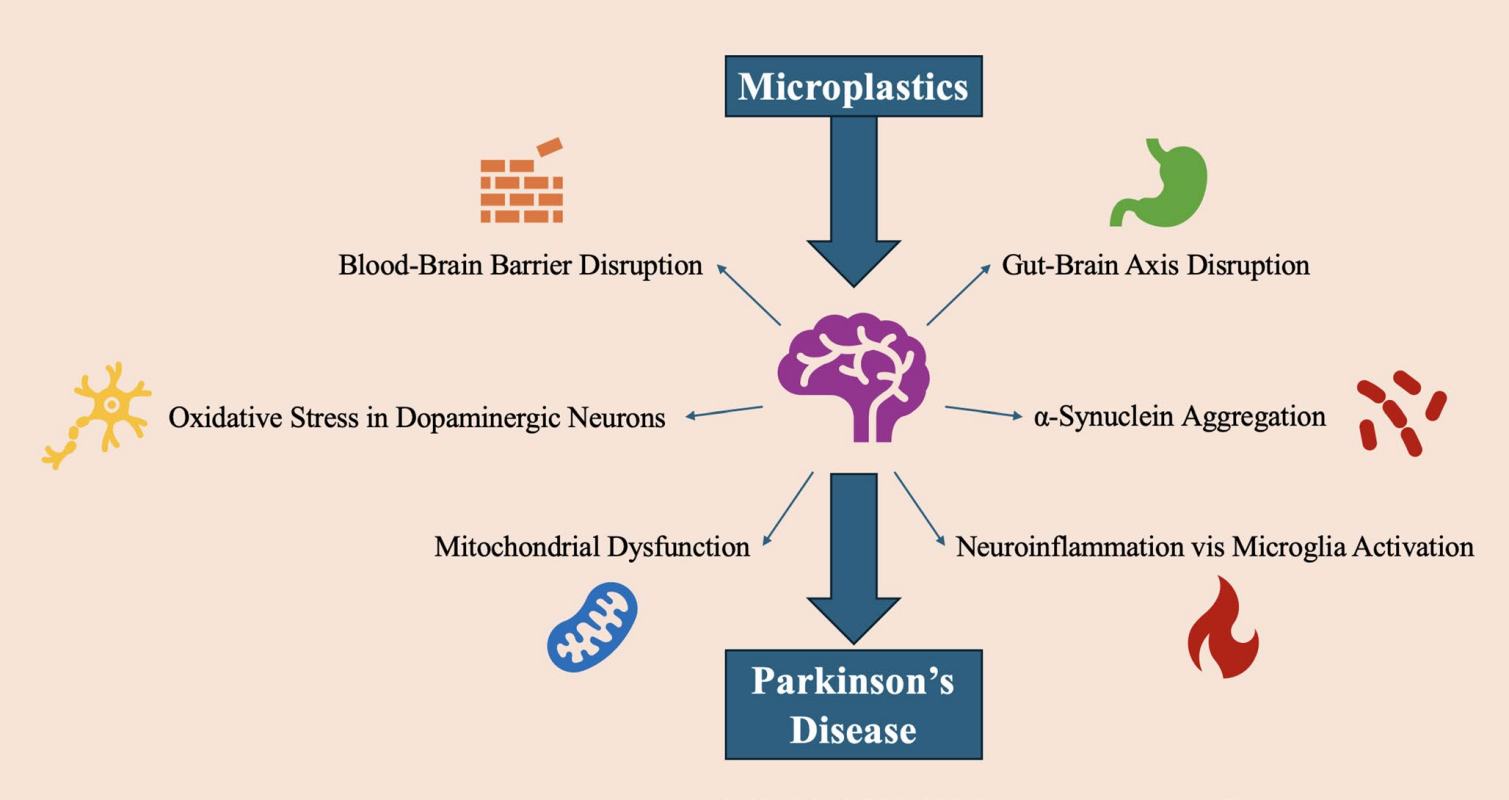
Potential Mechanisms for MPs in the development and progression of Parkinson’s Disease

MPs have been linked to the development and progression of PD. From our review of the current available literature on MP and PD, this review also found six possible mechanisms to explain MP’s role in PD development and progression. These include BBB disruption, oxidative stress in dopaminergic neurons, mitochondrial dysfunction, chronic neuroinflammation via microglial activation, α-synuclein aggregation, and Gut-Brain Axis [2] disruption (Fig. 7). Further, as some of the potential mechanisms have already been discussed, this section will go into more detail on other potential mechanisms more specific to PD.

### Blood-brain barrier disruption

Evidence indicates that MPs can cross and damage the BBB by disrupting the expression and organization of tight junction proteins such as claudins, occludin, and ZO-1. This compromise in barrier integrity increases the brain and CNS’s exposure to both MPs and peripheral neurotoxicants, including pro-inflammatory cytokines and microbial-derived metabolites. Notably, as observed in AD, BBB dysfunction is an early pathological feature of PD, preceding substantial neurodegeneration [170]. Therefore, MPs may exacerbate BBB permeability, facilitating the infiltration of peripheral immune cells, activated microglia, and inflammatory mediators into the CNS. This neuroinflammatory environment may accelerate α-synuclein aggregation and contribute to dopaminergic neuronal loss in the substantia nigra—key hallmarks of PD.

### Oxidative stress in dopaminergic neurons

As previously discussed, MPs can induce the production of ROS, leading to elevated oxidative stress in the brain [145]. Dopaminergic neurons in the substantia nigra are particularly vulnerable to oxidative damage due to their high metabolic activity and the pro-oxidant nature of dopamine metabolism [171]. Oxidative stress disrupts the tightly regulated redox homeostasis within neurons, impairing multiple cellular processes and contributing to neuronal dysfunction and death [172]. Moreover, ROS can damage critical cellular components such as mitochondrial membranes and DNA, while also impairing ATP production, factors that collectively promote neurodegeneration [173]. Based on these findings, oxidative stress is a plausible mechanism by which MPs may contribute to the onset and progression of PD.

### Mitochondrial dysfunction

As previously discussed, MPs can impair mitochondrial dynamics by reducing membrane potential and promoting apoptosis [125]. Mitochondrial dysfunction is a well-established feature of PD, particularly in the early stages, with strong links to mutations in key genes such as *PINK1*, *Parkin*, and *DJ-1* [174]. Integrating these findings, MPs may induce mitochondrial dysfunction in neurons, thereby exacerbating PD pathogenesis. This hypothesis is supported by a 2022 study in which mice exposed to 0.25–250 mg/kg of PS-NPs over 28 days developed PD-like neurodegeneration [175]. Single-nucleus RNA sequencing of 62,843 brain nuclei revealed widespread cell-specific responses, particularly involving disrupted energy metabolism and mitochondrial dysfunction across brain cells, with pronounced effects in excitatory neurons. Additionally, PS-NP exposure provoked inflammatory activation in astrocytes and microglia and disrupted proteostasis and synaptic regulation in oligodendrocytes, astrocytes, and endothelial cells—mechanisms closely associated with PD. Notably, PS-NPs targeted key PD-related brain regions, including the substantia nigra pars compacta and striatum, leading to decreased ATP levels and downregulation of ATP-associated genes and proteins. Liang et al. investigated that PLA polymer and oligomer microplastics’ toxicity, biodistribution, and gastrointestinal breakdown in mice were examined. An incomplete breakdown of PLA polymers resulted in oligomer nanoplastics with higher toxicity and bioavailability, whereas full degradation of PLA oligomers decreased toxicity. Both kinds caused neurotoxicity in the midbrains that resembled Parkinson’s disease through MICU3-mediated mitochondrial calcium excess, which could be reduced by adjusting mitochondrial calcium flow. These results demonstrate how biodegradable microplastics may pose health hazards to people [176]. Collectively, these findings underscore the mechanistic potential of MPs in driving PD onset and advancement.

### Chronic neuroinflammation via microglial activation

MPs have been shown to induce chronic neuroinflammation through the activation of microglia [110]. Notably, prolonged microglial activation exerts selective neurotoxic effects on dopaminergic neurons, which are particularly susceptible due to their high oxidative burden and limited antioxidant defenses [177]. Chronic neuroinflammation is a hallmark feature of PD, contributing to synaptic dysfunction, neuronal loss, and disease progression [178].

As previously noted, activated microglia release pro-inflammatory cytokines such as TNF-α and IL-1β, as well as ROS, all of which contribute to heightened neuronal stress and facilitate the propagation of α-synuclein [110, 179]. Additionally, neuroinflammation has been shown to impair the clearance of α-synuclein, further promoting its accumulation and toxicity. Together, these processes suggest that MP-induced microglial activation may initiate or exacerbate neuroinflammatory cascades, ultimately increasing dopaminergic neuron loss and contributing to the development and progression of PD.

Furthermore, MPs have been shown to activate the NLRP3 inflammasome, primarily through ROS generation, lysosomal damage, and the release of danger-associated molecular patterns (DAMPs) [142]. Activation of NLRP3 promotes the secretion of pro-inflammatory cytokines such as IL-1β and IL-18, intensifying neuroinflammation. Importantly, NLRP3 has been closely linked to PD pathogenesis, as its activation contributes to both α-synuclein accumulation and neuronal cell loss [180], further highlighting the potential role of MPs in driving PD-related neurodegeneration.

### α-Synuclein aggregation

At the cellular level, MPs—particularly anionic NPs—have been shown to promote the misfolding and aggregation of α-synuclein, a key protein involved in PD pathogenesis. In a study by Liu et al. (2023), anionic NPs accelerated α-synuclein fibril formation and propagation via high-affinity interactions with the amphipathic and non-amyloid component (NAC) domains of α-synuclein. These interactions not only initiated fibril formation but also amplified the spread of α-synuclein pathology across connected, vulnerable brain regions. Notably, this effect was especially pronounced in dopaminergic neurons of the substantia nigra, a hallmark region affected in PD [181].

Based on these findings, MPs may induce α-synuclein aggregation within dopaminergic neurons of the substantia nigra, contributing to PD onset and progression. This is further supported by Jeong et al. (2024), who demonstrated that NPs exacerbated PD-like symptoms in both human cells and *C. elegans*, including motor dysfunction, accumulation of α-synuclein aggregates, and dopaminergic neuron degeneration [182]. Additionally, a 2022 study by Liang et al. found that polystyrene NPs influenced the formation of NACore oligomers, considered surrogates for α-synuclein aggregates and strongly implicated in PD pathology [175]. These data provide a mechanistic link between MPs and protein misfolding, reinforcing our hypothesis that MPs can drive PD-like neurodegeneration via α-synuclein aggregation.

Furthermore, Liu et al. revealed that NPs are internalized into neurons through clathrin-mediated endocytosis, which leads to lysosomal dysfunction and reduced degradation of aggregated α-synuclein. Impairments in autophagy, a critical protein-clearance pathway, are well-documented contributors to PD. Dysfunctional autophagy hinders the removal of misfolded proteins and fosters α-synuclein accumulation, eventually promoting Lewy body formation due to α-synuclein’s prion-like spreading behavior [183, 184]. Thus, MPs may impair lysosomal and autophagic pathways, providing a plausible mechanism for PD pathogenesis and progression through α-synuclein accumulation and dopaminergic neuron loss.

### Gut-brain axis disruption

MPs have been implicated not only in disrupting brain function but also in interfering with the gut microbiota [185]. Their impact on the gut microbiome occurs via three primary mechanisms: physical disruption, chemical interactions, and environmental alterations within the gastrointestinal (GI) tract. Physically, MPs can damage the intestinal lining, increasing intestinal permeability (commonly known as “leaky gut”) and disturbing microbial communities by altering their structure, growth, survival, and metabolic activity. For example, animal studies have shown that MP exposure is associated with an enrichment of *Firmicutes*, *Proteobacteria*, and *Chlamydia*, alongside a reduction in *Bacteroidetes* populations [186]. Chemically, MPs can induce oxidative stress and inflammation in the gut, further disrupting microbial homeostasis [187]. Together, these mechanisms contribute to significant changes in microbial diversity, composition, and function, culminating in a state of dysbiosis [185].

The gut–brain axis [2] is a bidirectional communication system connecting the GI tract and the CNS, primarily via the vagus nerve [111]. This axis plays a crucial role in maintaining systemic homeostasis, influencing immune, neural, and metabolic processes. The gut microbiota is a key regulator of GBA signaling, largely through the production of metabolites and neurotransmitters [188]. For instance, alterations in the gut microbiota can affect the synthesis of neurotransmitters and neuromodulators (e.g., serotonin and dopamine), production of short-chain fatty acids (SCFAs) such as acetate, which help regulate neuroinflammation, and the development and modulation of immune responses. As such, disturbances in the gut microbiota, particularly dysbiosis, can impair gut–brain communication and contribute to neurological dysfunction.

Dysbiosis has been increasingly associated with both systemic and neuroinflammation. The compromised integrity of the gut barrier allows microbial products and endotoxins to translocate into the bloodstream, triggering systemic inflammation [189]. Additionally, the release of pro-inflammatory molecules like lipopolysaccharides (LPS), along with reductions in key neurotransmitters such as serotonin, can activate inflammatory responses in the brain [190, 191].One of the study examined the relationship between intestinal health and neurotoxicity by examining how oral exposure to 50 nm PS-NPs alters the gut microbiota in mice, raising IL-17 C levels that reach the circulation and cause inflammation in the brain. Antibiotics or anti-IL-17 C antibodies reduced the neurotoxic effects [192]. Dysbiosis also impacts immune regulation, as imbalances in microbial populations can lead to altered immune cell activity, chronic inflammation, and subsequent neuroinflammatory cascades [193].

In the context of PD, dysbiosis has been linked to the production and aggregation of α-synuclein, a hallmark protein in PD pathogenesis. Systemic inflammation driven by gut dysbiosis can activate the enteric nervous system and induce the release of cytokines that promote α-synuclein accumulation within enteric neurons [194, 195]. Moreover, dysbiotic gut environments may enhance the production of bacterial amyloid proteins that act as scaffolds, facilitating the misfolding and aggregation of α-synuclein [196]. These aggregates can then travel along the vagus nerve to the brain, potentially initiating or accelerating the progression of PD. In particular, α-synuclein aggregates have been shown to propagate retrogradely along the vagus nerve to the dorsal motor nucleus of the vagus in the brainstem, a proposed early site of PD pathology [197].

Based on current literature, this review proposes that MPs may contribute to PD pathogenesis by inducing gut dysbiosis. This hypothesis is further supported by clinical observations that PD patients often exhibit an increased abundance of potentially harmful bacteria and a reduction in beneficial microbial populations [198]. These findings align with the Braak hypothesis, which suggests that PD pathology originates in the gut and spreads to the brain through the enteric nervous system or vagus nerve in a prion-like manner [199]. However, many of these studies are currently conducted in animal models at high MP concentrations, and therefore, many of these findings are difficult to extrapolate.

## Limitations

A key limitation of our study on MPs and their roles in AD and PD is the disproportionate focus on PS-MPs and PS-NPs. This imbalance stems from the overwhelming number of studies conducted using PS-MPs/NPs, largely due to their commercial availability, cost-effectiveness, modifiable surface chemistry, and low variability [12, 14]. However, as previously noted, PS is only the third most commonly used polymer globally, comprising 9.7% of plastic production [12], and ranks as the fourth most prevalent MP type in aquatic environments (8.5%) [13]. Consequently, our review overrepresents PS-MPs and does not adequately reflect more abundant MPs in aquatic systems, such as polyethylene (25%), polyethylene terephthalate (16.5%), and polypropylene (14%). Moreover, while PS-MPs/NPs dominate the literature, other polymers such as PE, PP, and PET may exhibit distinct bioaccumulation, transport, and neurotoxic profiles. In addition, variations in particle shape, including fibers, fragments, and beads, can influence interactions with biological systems. Expanding research to include a broader range of polymers and morphologies is necessary to generalize conclusions about microplastic-induced neurodegeneration.

Environmental toxicants such as Bisphenol-A (BPA), its analogs (BPF and BPS), and phthalates highlight convergent mechanisms of oxidative stress, mitochondrial dysfunction, apoptosis, and impaired neurogenesis. For example, BPF and BPS exposure reduce neural stem cell proliferation, neuronal differentiation, and synaptic integrity while increasing apoptosis in hippocampal cells, ultimately impairing cognition. Similarly, epidemiological analyses link phthalates and BPS exposure to amyloid-β positivity and mild cognitive impairment/Alzheimer’s disease, suggesting that these compounds may accelerate neurodegenerative processes [200]. Although some studies indicate that BPS may lack significant hepatotoxicity and may not cross the blood-brain barrier acutely, its long-term neurological safety remains unclear [201]. These findings parallel mechanisms described for CBD-induced oxidative stress modulation and microplastic-associated neurotoxicity, where mitochondrial dysfunction and disrupted calcium signaling drive neuronal injury. Together, this body of evidence underscores the importance of investigating shared molecular pathways—oxidative stress, mitochondrial impairment, apoptosis, and disrupted neurogenesis—that may contribute to the pathogenesis of neurodegenerative disorders such as Alzheimer’s and Parkinson’s disease [202–204].

Another major limitation of our review is the scarcity of studies directly linking MP exposure to neurodegeneration, particularly in relation to AD and PD in humans. At present, most available research is preclinical, relying on in vitro models or animal studies. While these provide valuable mechanistic insights, they fall short of establishing clear connections and conclusions between environmental MP exposure and neurodegenerative outcomes in humans. Compounding this issue is the lack of long-term studies investigating the effects of MPs on neurodegenerative disease development. Disorders such as AD and PD typically evolve over years or even decades [205], yet most MP studies are short-term—often limited to two or three weeks—and therefore may not capture the chronic impacts of prolonged exposure. Given the relatively recent emergence of MPs as an area of concern, there is an urgent need for longitudinal human studies to assess the cumulative effects of MP exposure, particularly in relation to neurological health.

## Future directions

While there is growing evidence that MPs may exacerbate or accelerate the progression of neurodegenerative diseases such as AD and PD, a direct causal relationship has not yet been established. To strengthen this link, longitudinal human cohort studies are essential [206]. These studies should monitor prolonged and low-dose MP contact and exposure through dietary intake, inhalation, and water sources, and correlate exposure levels with cognitive decline and early clinical signs of AD and PD. This methodology would more accurately represent actual environmental conditions and enable researchers to evaluate the long-term effects of microplastics on cognitive function [207]. Additionally, targeting high-risk populations, such as individuals living in heavily polluted regions, workers in plastic manufacturing, or those near waste disposal sites, could provide valuable insights into whether elevated MP exposure is associated with increased incidence of neurodegenerative disorders [208]. An example of such research could be a multi-year study that examines MP levels in biological samples, such as blood, stool, or cerebrospinal fluid, and correlates these findings with cognitive assessments and clinical biomarkers of neurodegeneration in plastic industry workers compared to control populations. This type of study would help elucidate the environmental contribution to MP exposure, provide valuable translational insights, and identify potential risk factors linked to neurological decline. At present, such longitudinal human studies represent the most urgent priority for clarifying the neurological risks of MP exposure. In addition, standardized in vivo models are needed to investigate the chronic effects of MP exposure on neurodegenerative diseases. For example, transgenic mouse models commonly used in AD (e.g., APP/PS1) and PD (e.g., α-synuclein overexpression) could be employed to study how prolonged MP exposure influences the onset and progression of neuropathology.

Building on these models, a promising research direction involves the development and validation of MP-specific biomarkers, particularly in human biological samples (e.g., blood, stool, cerebrospinal fluid), which would allow for direct translational relevance. Identifying markers such as oxidative stress indicators and neuroinflammatory cytokines could help differentiate MP-induced neurotoxicity from other environmental or genetic triggers [209]. Additionally, biomarkers of oxidative stress (e.g., ROS, superoxide dismutase [SOD], and catalase [CAT]), inflammation (e.g., TNF-α), and genotoxicity (e.g., micronuclei and chromosomal aberrations) should be validated in standardized models and human studies to confirm their reliability. While pharmacological or nutritional interventions (e.g., antioxidants, anti-inflammatory compounds) may eventually hold promise, these investigations should follow after establishing causal links in humans and standardized MP models. Currently, the lack of standardized MP models and protocols remains a critical barrier [210]. Future research should prioritize developing harmonized methodologies for particle type, size, surface chemistry, and environmentally relevant dosing. This standardization will enable reproducibility and cross-study comparisons, and is essential before firm conclusions about human health risks can be drawn.

## Conclusion

In this review, the current literature is synthesized to explore the potential mechanisms linking MP exposure to the development of neurodegenerative diseases, specifically AD and PD. Our analysis suggests that MPs may contribute to neurodegeneration through several interconnected pathways, including the activation of resident immune cells in the brain (e.g., microglia and astrocytes), induction of oxidative stress, disruption of the BBB, mitochondrial dysfunction, and direct neuronal damage. The degree to which they contribute to the onset and progression of the disease in humans remains uncertain. For AD, six major mechanistic pathways were identified: BBB disruption, chronic inflammation, oxidative stress and ROS generation, mitochondrial dysfunction, impaired autophagy and proteostasis, and epigenetic modifications with gene dysregulation. Similarly, six key mechanisms were associated with PD: BBB disruption, oxidative stress in dopaminergic neurons, mitochondrial dysfunction, microglial-driven neuroinflammation, α-synuclein aggregation, and GBA disruption. These overlapping mechanisms underscore the multifactorial and self-perpetuating nature of MP-related neural stress, though much of the evidence remains preliminary and derived from in vitro and animal models.

Given the growing global population and the persistent use of plastics in daily life, there is an urgent need for further research into MP-related neurological outcomes. Long-term, standardized studies are needed to clarify causality, validate mechanistic pathways in vivo, and establish the extent of MPs’ contribution to AD and PD. Such research will not only inform potential preventive and therapeutic approaches but also provide critical evidence for environmental and public health policies for MP-associated AD and PD. Moreover, these insights can support environmental policy initiatives aimed at reducing plastic production, improving waste management, and mitigating the far-reaching public health risks posed by this pervasive and silent environmental contaminant.

## Data Availability

No datasets were generated or analysed during the current study.
